# A Single-Chamber Microbial Fuel Cell without an Air Cathode

**DOI:** 10.3390/ijms13033933

**Published:** 2012-03-22

**Authors:** Vanita Roshan Nimje, Chien-Cheng Chen, Hau-Ren Chen, Chien-Yen Chen, Min-Jen Tseng, Kai-Chien Cheng, Ruey-Chyuan Shih, Young-Fo Chang

**Affiliations:** 1 Department of Life Science, National Chung Cheng University, 168 University Road, Minhsiung, Chiayi, 621, Taiwan; E-Mails: vanita.nimje@gmail.com (V.R.N.); biohrc@ccu.edu.tw (H.-R.C.); biomjt@ccu.edu.tw (M.-J.T.); 2 Department of Biotechnology, National Kaohsiung Normal University, No. 62, Shenjhong Rd., Yanchao Township, Kaohsiung County, 82444, Taiwan; E-Mail: xavierch2000@yahoo.com; 3 Department of Earth and Environmental Sciences, National Chung Cheng University, 168 University Road, Minhsiung, Chiayi 621, Taiwan; E-Mails: cheng.168@ccu.edu.tw (K.-C.C.); seirock@eq.ccu.edu.tw (R.-C.S.); seichyo@eq.ccu.edu.tw (Y.-F.C.); 4 Department of Engineering, University of Cambridge, Trumpington Street, Cambridge CB2 1PZ, UK

**Keywords:** microbial fuel cells, *Bacillus subtilis*, cyclic voltammograms, nitrate reduction, air cathode, glucose, fermentation, microbial growth, aerobic

## Abstract

Microbial fuel cells (MFCs) represent a novel technology for wastewater treatment with electricity production. Electricity generation with simultaneous nitrate reduction in a single-chamber MFC without air cathode was studied, using glucose (1 mM) as the carbon source and nitrate (1 mM) as the final electron acceptor employed by *Bacillus subtilis* under anaerobic conditions. Increasing current as a function of decreased nitrate concentration and an increase in biomass were observed with a maximum current of 0.4 mA obtained at an external resistance (*R*_ext_) of 1 KΩ without a platinum catalyst of air cathode. A decreased current with complete nitrate reduction, with further recovery of the current immediately after nitrate addition, indicated the dependence of *B. subtilis* on nitrate as an electron acceptor to efficiently produce electricity. A power density of 0.0019 mW/cm^2^ was achieved at an *R*_ext_ of 220 Ω. Cyclic voltammograms (CV) showed direct electron transfer with the involvement of mediators in the MFC. The low coulombic efficiency (CE) of 11% was mainly attributed to glucose fermentation. These results demonstrated that electricity generation is possible from wastewater containing nitrate, and this represents an alternative technology for the cost-effective and environmentally benign treatment of wastewater.

## 1. Introduction

Energy generation from the treatment of wastewater is of great interest due to the worsening global energy crisis. Industrial and agricultural operations produce large amounts of wastewater, the treatment of which is costly. Wastewater from agricultural operations contains high levels of nutrients in the form of nitrates and phosphates, which can pollute groundwater and surface water through eutrophication [1]. Before being released into the environment, it is obligatory to treat wastewater to meet discharge regulations. Biological denitrification is a wastewater treatment process facilitated by microorganisms and is considered the most reliable and cost-effective nutrient removal process [2,3].

Electricity generation using microbial fuel cells (MFCs) with denitrification has drawn considerable attention in recent years as a new approach to wastewater treatment. In MFCs, bacteria at the anode consume organic matter through metabolism and generate electrons and hydrogen ions [4]. Electron migration from anode to cathode occurs through the external circuit, and protons migrate in the solution from anode to cathode to form water. Biological denitrification is the reduction reaction towards the cathode, which is represented by [5]:

(1) $${{\text{NO}}_{3}}^{-}+6{\text{H}}^{+}+5{\text{e}}^{-}\to 1/2{\text{N}}_{2}+3{\text{H}}_{2}\text{O}$$

In this process, microorganisms at the cathode reduce nitrate by accepting the electrons released by substrate oxidation at the anode. Hence, this method of wastewater treatment and electricity generation in MFCs has received considerable attention in recent years due to the simultaneous removal of contaminants such as nitrogenous waste and energy generation. The cathode plays an important role in the consumption of electrons by reducing electron acceptors such as nitrate [6]. Recently, there has been an increased interest in replacing abiotic cathodes with biocathodes in which microorganisms enhance the reduction reaction. A graphite cathode, a biocathode and a platinum-coated cathode have been used for denitrification [7]. A study conducted by Clauwaert *et al.* with respect to bioelectrochemical system applied microbial biocathodes using bacteria as the biocatalyst and nutrient-rich synthetic wastewater with acetate (electron donor) and nitrate (electron acceptor) [3]. Recently, Sukkasem *et al.* also reported the effect of nitrate concentration on electricity generation in a single-chamber MFC with a platinum-coated air cathode [5]. In that study, nitrate and oxygen both were used as the electron acceptors, which might have resulted in the bacterial community sustaining a non-electricity generating process. In addition, the use of an air cathode in single-chamber MFCs could also lower the coulombic efficiency (CE), as oxygen diffusion through the cathode to the anode disturbs the anaerobic conditions of the MFC and also provides an alternative acceptor to the bacteria. In order for bacteria to produce a current, the cells must use the anode as an electron acceptor, and involve no other electron acceptors such as oxygen. Therefore, microorganisms in an MFC are typically grown under completely anoxic conditions.

Recently, a new concept of floating-type microbial fuel cell (FT-MFC) has been introduced for extracting energy from water bodies in which oxygen is almost depleted due to high levels of organics contamination. In FT-MFC, the anode is located in the organics-contaminated water phase and a part of the cathode is exposed to the atmosphere and it was demonstrated that it is possible to directly convert organics in the water phase to electricity using the FT-MFC [8]. The second system, applied to water bodies contaminated by organic waste, is a multiphase electrode MFC (multiphase MFC)—A combination of an FT-MFC and a sediment MFC. Using these MFC systems, when temporal or consistent organic pollution has occurred in water bodies, it is possible to harvest a current by utilizing the organic materials that coexist in water and sediment phases [9]. Another study has shown that the crossover of organic compounds (substrates and metabolites) from the anode compartment of an MFC to the cathode may produce electric current. Substrate crossover in membraneless MFC considerably decrease the electrode performance. But due to the formation of mixed potentials and the flow of internal currents, electricity can be generated [10].

Microorganisms with different physiologies, such as *Escherichia coli*, *Geobacter sulferreducens*, *Enterobacter cloacae* and *Bacillus subtilis*, have been shown to generate electricity in MFCs [11–13]. It has been reported that *B. subtilis* can grow anaerobically using either nitrate or nitrite as a terminal electron acceptor, but the use of *B. subtilis* as a pure culture with simultaneous nitrate reduction in MFCs has not yet been exploited [14].

**Scheme 1:**
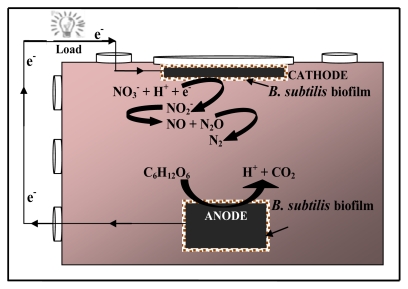
Schematic of the nitrate reduction process with *Bacillus subtilis* in a single chamber microbial fuel cell.

Taking the above consideration into account, in this study, a single-chamber MFC was constructed without oxygen and platinum cathode; carbon cloth was used as the anode and cathode, and the effect of glucose and nitrate on electricity generation by *B. subtilis* in the single-chamber MFC was analyzed, as shown in Scheme 1. We demonstrated a bioelectrochemical system that utilizes glucose as the carbon source and nitrate as an electron acceptor for *B. subtilis* that could account for electricity generation and simultaneous nitrate reduction in a single-chambered MFC without air cathode. The CE and polarization curve were obtained and cyclic voltammetry was conducted to evaluate the performance of the MFC.

## 2. Results and Discussion

### 2.1. Nitrate Respiration and Current Generation

**Figure 1:**
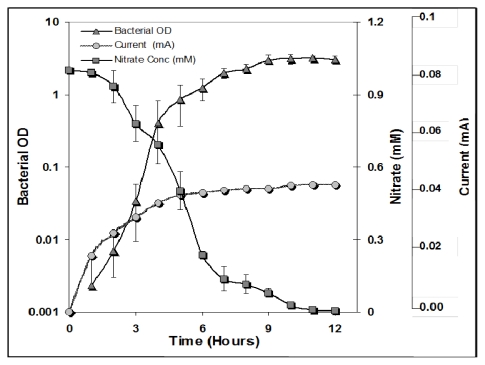
Growth of *Bacillus subtilis* (BBK006), wild-type ( 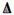) with nitrate reduction ( 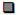) and an increase in current ( 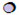). The concentrations given were determined at the indicated time points in the *Bacillus subtilis* cultures with 1 mM nitrate and 1 mM glucose. The error bars indicate the standard deviation based on the average of three separate experiments.

In order to investigate the growth of *B. subtilis* with nitrate as an electron acceptor, 1 mM nitrate and 1 mM glucose were provided in the experiments. Figure 1 shows the nitrate reduction and current generation *versus* bacterial growth. It could be observed that by utilizing glucose for oxidation and nitrate for reduction, *B. subtilis* grew efficiently. Although, we did not measured glucose concentration, we assume that as a result of glucose oxidation, liberated electrons were utilized in nitrate reduction with the concomitant decrease in nitrate concentration. During this period, planktonic growth in the reactor was also increasing as measured by the optical density (OD) values. Figure 1 demonstrates that a decrease in nitrate concentration resulted in an increase in current. The current reached a maximum of 0.05 mA within a period of around 12 hours (stationary phase), after which it started to cease as the nitrate concentration declined. Additionally, another 12 hours were required for the current to completely decrease. This might have been due to intermediates formed during nitrate reduction, which were slowly and steadily utilized by the bacteria, after which the current completely declined (data not shown). However, this value is much lower than those quoted in previous reports of *B. subtilis* nitrate reduction, possibly due to the use of Fe(III) EDTA as a mediator, enhancing electron transfer [2]. The low current generation (0.05 mA) observed initially lasted only for the first few hours, which could be attributed to the length of time required for the bacteria to adapt to an alternate electron acceptor and biofilm formation. This result implied that *B. subtilis* was involved in electron transfer from anode to cathode, with nitrate as an electron sink. These results corroborate those found by Park *et al.* [15], in which added Fe(III) served as an electron sink for the growth of *Shewanella putrefaciens* IR-1 and MR-1 strains in the electron transport system.

### 2.2. Effect of Nitrate Addition During Different Batch Cycles and Its Relationship with Current Generation and Nitrate Reduction

**Figure 2:**
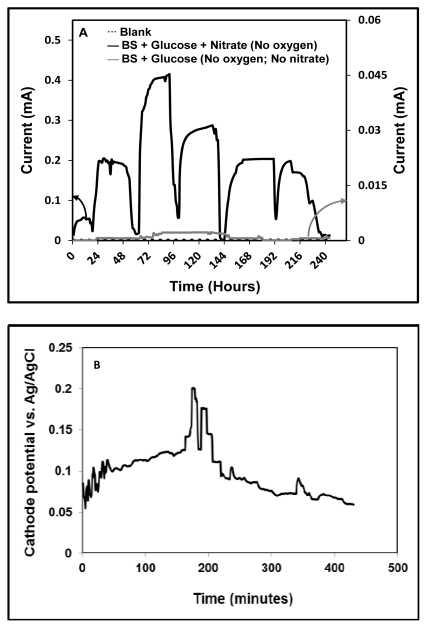
(**A**) Current generation from the microbial fuel cell with a 1-KΩ *R*_ext_ Blank (……), glucose-fed ( 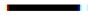), and glucose with nitrate-fed ( 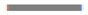); (**B**) Cathode half-cell potential recorded at the end of the experiment.

In order to investigate the effect of nitrate in terms of enhancing the current generation from *B. subtilis*, experiments were conducted in two sets with two different growth conditions in different reactors. The first set of experiments (MFC1) included the addition of glucose after inoculation of *B. subtilis* (no nitrate); in the second set of experiments (MFC2), glucose and nitrate were both added after inoculation of *B. subtilis*. Figure 2A shows the profiles of the cycles of current generation by the MFC under the two different conditions. Figure 2B shows the cathode half-cell potential recorded for MFC2. The cathode half-cell potential of the reactor was measured by placing an Ag/AgCl reference electrode on the cathode side of the MFC. For the first set of experiments (no nitrate), *B. subtilis* was unable to metabolize the substrate glucose due to the absence of terminal electron acceptors. However, the generation of an ultra-low current (0.002 mA) might have been due to glucose fermentation, which generates sufficient ATP for growth using substrate-level phosphorylation [16–18]. In the second set of experiments, six cycles of current generation were carried out using glucose and nitrate as the electron donor and acceptor, respectively. Corresponding to the addition of glucose and nitrate in individual cycles, a maximum current of 0.4 mA was observed, and all cycles lasted for around the same duration. After reaching the maximum value, the current declined thereafter, presumably due to the physiological and metabolic variations of the microbial populations in the MFC (second set of experiments). The current began to increase and again reached a peak after the MFC was re-fed with 1 mM glucose and 1 mM nitrate. Although the glucose concentration was not measured, whenever the nitrate concentration decreased, a sharp decrease in the current was also observed, which was followed by the addition of both nitrate and glucose. When the nitrate concentration started to decrease, the current was found to have reached its peak (around 0.5 mM nitrate was consumed—Data not shown), and subsequently declined until the end of the batch (around 0.05–0.1 mM nitrate remained—Data not shown). After the peak in the current, the slow declination might be attributed to the reduction of further intermediates that might be formed during nitrate reduction, which was slowly and steadily reduced by the bacteria, after which complete current diminution resulted. There was no current generation without bacteria, as verified by the control experiment. In the set of experiments in which only glucose was fed, the current output was quite low, owing to the lack of nitrate as an electron sink. This difference in current generation clearly indicates the significance of nitrate as a terminal electron acceptor for current generation in this study. We also conducted an experiment utilizing an air cathode for denitrification (data not shown). We observed that, in the presence of oxygen, *B. subtilis* was inefficient in reducing nitrate. Since oxygen has a higher potential for reduction relative to nitrate, if dissolved oxygen (through air cathode) is present, it will be reduced by the bacteria via aerobic respiration, resulting in the consumption of substrate and aerobic biomass growth without electricity generation. We presume that oxygen might have served as a dominant electron acceptor compared to nitrate in this system, which influenced the nitrate reduction in the air cathode MFC. A study conducted by Nakano *et al.* claims that there are two modes of anaerobic growth of *B. subtilis*: (a) by anaerobic respiration using nitrate as an electron acceptor and (b) by glucose fermentation [19]. Although *B. subtilis* has been reported to be a glucose fermentation strain, assumptions based on our experiments indicate that *B. subtilis* adopts both modes—Anaerobic growth with nitrate respiration and a fermentative pathway for glucose breakdown. In addition, at the end of each batch cycle, plenty of gas bubbles were observed, which indicated the possibility of conversion of nitrate into dinitrogen gas (N_2_).

In a similar study by Sukkasem *et al.*, the effect of nitrate on the performance of a single chamber air cathode MFC was conducted. It was found that no electricity was produced when the cell was moved to an anaerobic glove box (where no oxygen was present). Whereas formation of gas bubbles, presumably due to the removal of nitrate in the form of dinitrogen gas, was also observed as an end product of the denitrification process [5]. These results differ from those presented by Sukkasem *et al*., who found no evidence for electricity production in an anaerobic glove box. We believe that in our study, nitrate as an electron acceptor may not be able to compete with oxygen, and due to the aerobic respiratory pathway, oxygen is the preferred electron acceptor for *B. subtilis*. The electricity generation resulting from the use of nitrate in a single-chamber MFC is a proof-of-concept demonstration of a technology that links MFCs with the denitrification process in wastewater treatment.

### 2.3. Polarization Curve

**Figure 3:**
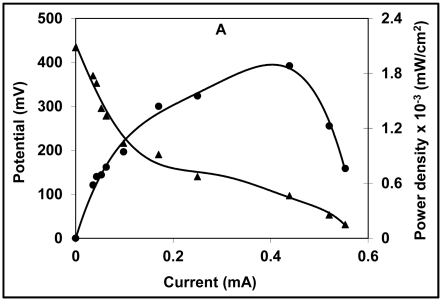

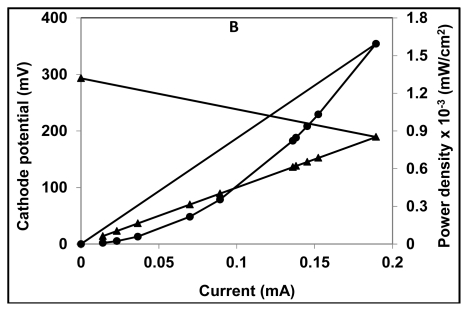
(**A**) Polarization curve with open circuit potential (OCP, ▴) and power density (●) measurements at variable external resistance (*R*_ext_) between 0.056 KΩ and 10 KΩ. The left axis shows the open circuit voltage OCV as a function of current and the right axis represents the resulting power density; (**B**) Polarization data for cathode half-cell.

Polarization curves are used for the analysis and characterization of fuel cells, and the relationship between cell voltage and current can be obtained from a polarization curve. When the external resistance (*R*_ext_) is infinite, *i.e.*, under open circuit conditions, no current flows and the open circuit voltage (OCV) is obtained. Contrary to this, when the *R*_ext_ is zero, the short circuit current is obtained. The power performance curve can also be calculated from the polarization curve, which represents the relationship between power generation and a given current. Figure 3A,B shows the polarization curve as a function of current, potential and power density measured at a variable *R*_ext_ (10–0.056 KΩ). At an infinite *R*_ext_, an OCV of 433 mV was obtained. Dominance of activation loss was observed from an initial steep decrease in the voltage from an OCV of 433 mV to 366 mV [20,21]. Voltage stabilization was comparatively rapid at the higher resistances studied. The subsequent slope of the voltage decreased from 0.17 mA to 0.5 mA and was almost linear, which might indicate the dominance of ohmic loss. The relatively lower drop in voltage and maximum current obtained at lower resistances reveals a lower potential drop and lower mass transfer losses at the electrode. The effective electron discharge observed at lower resistances is a probable reason for the further potential drop and slow stabilization of the voltage at lower resistances. Current generation with different *R*_ext_ was measured once the maximum voltage was attained. The curve depicts a maximum power density of 0.0019 mW/cm^2^ (0.44 mA) at the lower resistance (0.22 KΩ) studied. Current generation showed a decreasing trend with an increase in *R*_ext_, which is consistent with the literature, and indicated typical fuel cell behavior. At a higher *R*_ext_ (10 KΩ), a relatively lower power density of 0.0006 mW/cm^2^ (0.036 mA) was observed. The power produced by the system is limited due to the high internal resistance (*R*_int_) of 0.86 KΩ. The high *R*_int_ obtained in our study is still a subject of investigation. However, oxidation of substrates by microbes was observed to be greater at lower resistances than at higher resistances, where microbes donated electrons to the anode as the electrons were discharged in a closed circuit. Figure 3B shows a sudden drop in the potential might at lower resistance might be due to substrate exhaustion because of which electrons are unable to reach at the cathode. Higher polarization/electron transport resistance and domination of other electrochemical losses (e.g. activation, ohmic and polarization) are other possible factors that could result in the sudden voltage drop.

### 2.4. Electrochemical Activity as a Result of Change in Electron Acceptor

**Figure 4:**
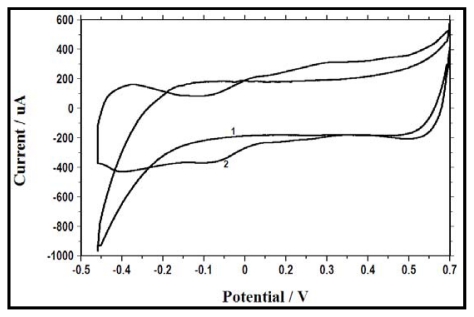
Cyclic voltammograms (CV) for the first set (1 − *Bacillus subtilis* + 1 mM glucose) and second set (2 − *Bacillus subtilis* +1 mM glucose + 1 mM nitrate) of experiments after inoculation of *Bacillus subtilis*.

Cyclic voltammetry (CV) measures the redox reaction and catalytic processes occurring at the electrodes. In this study, to determine the electrochemical activity, voltammograms were recorded and analyzed from the two sets of experiments described in the section above (in the first set of experiments, inoculate *B. subtilis* and glucose were added (no nitrate); in the second set of experiments, inoculate *B. subtilis* and glucose and nitrate were added). Initially, voltammograms were obtained for the first set of experiments. Following bacterial inoculation and the addition of glucose, at time zero, no redox peak was observed, as can be seen in Figure 4 (1 − glucose + *B. subtilis*). The addition of nitrate resulted in electrochemical activity of *B. subtilis*, wherein two reduction peaks at about −0.1 V and −0.4 V (*vs.* Ag/AgCl) and two oxidation peaks at about 0.05 V and 0.25 V (*vs.* Ag/AgCl) were observed at time zero (Figure 4: 2 − glucose + nitrate + *B. subtilis*). This two-step change in the peak potential indicates the occurrence of a two-electron transfer mechanism, which reveals the presence of two redox species in the solution or at the surface of the anode that are reversibly oxidized or reduced during the CV tests, resulting in current generation [22,23]. This result also suggests that current generation by the MFC might depend on the electrochemical activity of the bacteria: the redox peaks with catalytic current were significantly higher in all potential regions when the medium contained glucose, nitrate (MFC2) and *B. subtilis* in comparison with the voltammograms obtained using only glucose and *B. subtilis* (MFC1). These electrochemically-active compounds could be responsible for the increase in current production following nitrate addition.

### 2.5. Electrochemical Activity During Different Fed Batch Cycles and Electron Transfer Mechanisms

**Figure 5:**
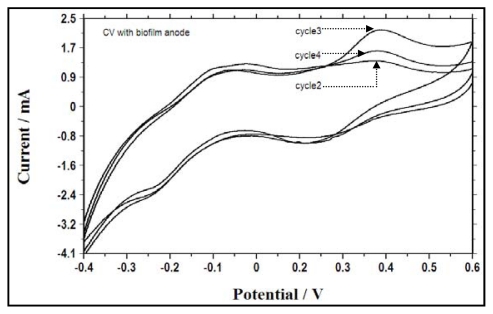
Cyclic voltammograms of the bacterial culture with a biofilm anode in batch cycles 2, 3 and 4.

**Figure 6:**
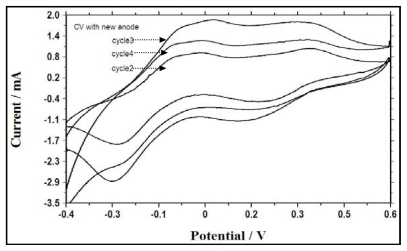
Cyclic voltammograms of the bacterial culture with a new anode in batch cycles 2, 3 and 4.

In order to investigate the correlation between electrochemical activity at different batch cycles and associated electron transfer mechanisms, cyclic voltammograms were recorded and analyzed following the addition of glucose and nitrate in second, third and fourth batch cycles with the biofilm anode (Figure 5) and with a new anode (Figure 6). The shapes of the voltammograms showed no variance in the peak potential in different cycles, but different sizes were observed for the voltammogram obtained with the two-electron transfer mechanism showing two oxidation peaks (potentials of approximately −0.05 and 0.4 mV) and two reduction peaks (potentials of approximately 0.15 and −0.3 mV) (Figures 5 and 6). This indicated that the electrochemical activities of the bacteria in the reactor and the biofilm bacteria were similar, and the redox components responsible for the electrochemical activity reappeared in each batch cycle. It can be inferred that the reactant, *i.e.*, glucose, undergoes oxidation and produces another electrochemically-active species, with reconversion of the reduced species, *i.e.*, nitrate, back into its oxidized state, as the intensity of the reduction peak is quite high, which can be attributed to reduced species formed by electrochemical reaction in the reaction vessel [22]. However, we assume that during the nitrate reduction process other possible intermediates such as NO, N_2_O, and NO_2_ (although not analyzed) were formed, but reaction with other species in the reaction vessel could destroy them. A higher reductive current was obtained as the batch cycle progressed from the second cycle to the third cycle due to the increase in OD. Alternatively, from the third cycle to the fourth cycle, the current was found to decrease, implying spatial obstruction caused by the biofilm resulting in diffusion limitation between the solution and the anode.

Overall from these results, it can also be concluded that the electrochemical activities of the bacteria in the reactor and the biofilm bacteria were similar. Hence, it is believed that the electrochemical activity generated in the presence of the new anode was governed by the excreted metabolites. However, our results also implied that, in addition to the excreted metabolites, a direct electron transfer mechanism was also possible due to the attached bacteria (biofilm) at the anode. We postulate that *B. subtilis* mediate the electrons transfer towards the anode, either by excreting metabolic compounds into the reactor solution or by membrane-driven electron transfer. It is well known that *B. subtilis* possesses membrane-bound nitrate reductases; however, we presume that, in addition to the excreted metabolites, an extracellular direct electron transfer mechanism also operates with components associated with the bacterial cell wall/membrane-bound nitrate reductases. These results were in concurrence with those of Rabaey *et al.* [22], and highlighted the possibility of extracellular electron transfer through the production of extracellular shuttles or mediators, *i.e.*, metabolites, and components/enzymes associated with the bacterial cell membrane.

### 2.6. Performance of the MFC at the End of the Batch Test

**Figure 7:**
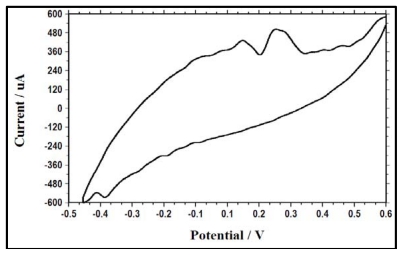
Cyclic voltammograms of the bacterial culture with a biofilm anode at the end of the batch test.

In the substrate depletion state, the voltammograms obtained showed very sharp oxidation peaks at potentials of 0.1 V and 0.2 V (*vs.* Ag/AgCl), as shown in Figure 7. This electrochemical activity shown by the bacterial cells is an indication of the presence of soluble redox-active compounds in the solution. Another possibility is that the activity may be due to the presence of mediators or metabolites that were stored in the cells, which maintained some storage power to utilize for their metabolic processes, even in the absence of a substrate [22].

### 2.7. Coulombic Efficiency (CE)

The overall performance of an MFC can be evaluated in many different ways. Principally, coulombic efficiency (CE) is used to compare the performances of MFCs in order to discuss the effect of the substrate used on electricity generation. However, several factors could contribute to the CE in MFCs, including oxygen diffusion through the cathode, an increase in the *R*_ext_, bacterial mediators [24] and biomass growth. The CE of the single-chamber MFC in the present study with a fixed *R*_ext_ of 1 KΩ was 11%. However, we assume that methanogenesis and/or fermentation are the most likely competitive and alternative metabolic pathways for the microbes, which could lower the CE. In our experiment, the neutral pH of the M9 medium remained stable over the course of the experiment, negating the possibility of methanogenesis. However, fermentation should be considered as an important factor for a reduced CE. Hence, in the present study, for a single-chamber MFC, a CE of 11% was obtained under more versatile conditions without the use of mediators, oxygen diffusion, proton exchange membrane (PEM), or a platinum cathode as compared with previous studies. Although complete conversion of the substrate to electrons was not achieved, MFC generating electricity with simultaneous nitrate reduction was developed in this study.

## 3. Experimental Section

### 3.1. Cultivation of Bacteria

To prepare the inoculum*, B. subtilis* BBK006 (wild-type) was grown in 25 mL of M9 medium [13]. The composition of the M9 medium used as the electrolyte in the MFC was as given by Miller [25]. The medium was sterilized at 121 °C for 20 min without glucose, which was filter-sterilized (Millipore membrane PVDF, 0.22-μm filter unit; Millipore, Watford, UK) and added to the fuel cell reactor after inoculation of the bacteria.

### 3.2. Microbial Fuel Cell Construction and Operation

The membrane-free single-chamber MFC consisted of an anode and a cathode placed in a plastic (Plexiglas) cylindrical chamber of 4 cm in length and 3 cm in diameter (empty bed volume of 28 mL), as reported previously [24,26]. The cathode was tightly packed with a plexiglass stopper. The inside surface of the carbon cloth was loaded with 4 PTFE diffusion layers without a platinum catalyst to avoid spontaneous passage or diffusion of solution pass-out from inside the MFC, the layers were prepared as described by Cheng *et al.* [26]. The exposed surface area of the anode electrode was 22.5 cm^2^ (dimension: 11.25 cm × 11.25 cm) and the diameter of the cathode was 3 cm. Titanium wire was used to connect the circuit, and the fuel cell was placed under constant load by connecting the cathode and anode to a *R*_ext_ of 1 KΩ. All experiments were conducted at room temperature. Following bacteria inoculation, the addition of 1 mM of nitrate and 1 mM of glucose achieved bacteria growth as well as nitrate reduction.

### 3.3. Biofilm Growth and Anaerobic Condition

Plain carbon cloth was used as anode and cathode for the development of biofilm. They were completely suspended in the bioreactor solution. The MFC reactor was placed vertically and the cathode was inoculated under aseptic anaerobic microenvironment on the top of the reactor at the solution boundary as shown in Scheme 1. Experiments were conducted to allow biofilm growth by inoculating bacteria and substrate addition of glucose and nitrate.

Anaerobic conditions in the reactor were maintained by the reduction or restriction of the amount of oxygen in the reactor. After inoculating bacteria into the MFC, bacteria were allowed to utilize oxygen present in the reactor solution as the terminal electron acceptor. During this period, ultra-low current was recorded. After the oxygen depletion (as measured by the dissolved oxygen concentration), current production almost dropped to zero, which implies an establishment of anaerobic conditions in the reactor. After which, we added glucose and nitrate for bacterial growth and energy production by the bacteria.

### 3.4. Optical Density Measurement

The bacteria were grown at optical density (OD_600_) of 1.55, and the final OD of the bacterial suspension in the reactor was determined spectrophotometrically. To measure OD from the MFC reactor, samples were taken from the vicinity of the electrode for accurate OD measurements. Hence, the OD values represent growth of planktonic bacteria in the MFC system.

### 3.5. Nitrate Measurement

Nitrate was determined spectrophotometrically by the sulphanylamide and *N*-(1-naphthyl)-ethylenediaminedihydrochloride method of Nicholas and Nason 1957 [27].

### 3.6. Data Acquisition and Electrochemical Technique

The electrode circuit potential and real-time data were continuously monitored and recorded by a computer, the anode and cathode having been connected directly to a PicoLog recorder using PicoLog^®^ v. 5.09.4 recorder software (Pico Technology Ltd., Cambridgeshire, UK) with an RS232 interface connected to an ADC 20–21 A–D converter (Pico Technology). The circuit was permanently connected to a 1 kΩ *R*_ext_ to obtain the anodic and cathodic potentials with respect to time, which were further used to obtain the current. To obtain the polarization curve, different *R*_ext_ were used: 10, 8.2, 6.8, 5.6, 4.7, 3.3, 2.2, 1, 0.56, and 0.22 kΩ.

Calculations: Potential (*V*) was monitored in order to calculate the current (*I*), as *I* = *V*/*R*. Power was normalized based on the cross-sectional area (projected area = 22.5 cm^2^) of the anode to calculate the power density in mW/cm^2^, as *P* = *I* × *V*/22.5 cm^2^. CE was calculated as *E*c = *C*p/*C*_Ti_100%, where *C*p (*C*) is the total Coulombs, calculated by integrating the current over time, and *C*_Ti_ (*C*) is the theoretical amount of Coulombs that can be produced from glucose (i = g), calculated as

$$C_{\text{Ti}}=F{\text{b}}_{\text{i}}{\text{S}}_{\text{i}}v/{\text{M}}_{\text{i}}$$

where *F* is Faraday’s constant (96,485 C/mole of electrons), b_i_ is the number of mol of electrons produced per mol of substrate (bg = 24 where, g represents glucose), S_i_ is the substrate concentration in g/L, v (L) is the liquid volume, and M_i_ is the molecular weight of the substrate (Mg = 180) [24].

### 3.7. Cyclic Voltammetry

Cyclic voltammetry was performed using a potentiostat (CHI 627C; CH instrument, Austin, TX, USA) connected to a personal computer (CHI627C Electrochemical Analyzer), with a scan rate of 1 mVs^−1^ ranging from −450 to 600 mV. Voltammograms were recorded using a conventional three-electrode set-up consisting of a working electrode, a reference electrode (an Ag/AgCl electrode), and a counter electrode (platinum wire). The working electrode was the carbon cloth anode with attached bacteria (*i.e.*, a biofilm anode) from the MFC reactor. For the purpose of voltammetry conducted without a biofilm anode, a new carbon cloth anode was employed as the working electrode (also with an electrode area of 22.5 cm^2^). The anode was washed with ethanol and deionized water prior to recording the voltammograms. *In situ* cyclic voltammetry was performed during different batch cycles to evaluate the electrochemical activity of the bacteria in progressive cycles. To investigate the possibility of any intermediate metabolic products being produced by the bacteria, voltammograms were obtained with and without a biofilm electrode.

## 4. Conclusions

Simultaneous denitrification and electricity generation was accomplished in a single-chambered MFC without air cathode, utilizing glucose as the substrate and nitrate as the terminal electron acceptor. We found that in a MFC in which oxygen diffusion through the cathode was restricted, a measurable current resulted when the single-chamber MFC was purposely maintained under anaerobic conditions for nitrate reduction at the cathode. A carbon/PTFE electrode without a platinum catalyst was found to be very suitable for use as the anode, successfully harvesting electricity from the bacterial metabolism, although glucose fermentation lowered the CE to 11%. In this study, we also found that the electrochemical activity of the bacteria was due to dissolved metabolic components secreted in the solution, along with a membrane-bound reductase system. The use of MFC technology to generate electricity and simultaneously treat nutrient-rich wastewater was demonstrated to be a feasible and attractive alternative energy system to expensive wastewater treatment processes. A single-chambered MFC is performed without the need of air cathode in which MFCs could be easily connected in series or in parallel for to amplification of the current and voltage production.
